# Comparative Analysis of the 5S rRNA and Its Associated Proteins Reveals Unique Primitive Rather Than Parasitic Features in *Giardia lamblia*

**DOI:** 10.1371/journal.pone.0036878

**Published:** 2012-06-07

**Authors:** Jin-Mei Feng, Jun Sun, De-Dong Xin, Jian-Fan Wen

**Affiliations:** 1 State Key Laboratory of Genetic Resources and Evolution, Kunming Institute of Zoology, Chinese Academy of Sciences, Kunming, People’s Republic of China; 2 Graduate School of Chinese Academy of Sciences, Beijing, People’s Republic of China; 3 College of Chemistry and Life Sciences, Zhejiang Normal University, Jinhua, People’s Republic of China; Charité-Universitätsmedizin Berlin, Germany

## Abstract

**Background:**

5S rRNA is a highly conserved ribosomal component. Eukaryotic 5S rRNA and its associated proteins (5S rRNA system) have become very well understood. *Giardia lamblia* was thought by some researchers to be the most primitive extant eukaryote while others considered it a highly evolved parasite. Previous reports have indicated that some aspects of its 5S rRNA system are simpler than that of common eukaryotes. We here explore whether this is true to its entire system, and whether this simplicity is a primitive or parasitic feature.

**Methodology/Principal Findings:**

By collecting and confirming pre-existing data and identifying new data, we obtained almost complete datasets of the system of three isolates of *G. lamblia*, two other parasitic excavates (*Trichomonas vaginalis*, *Trypanosoma cruzi*), and one free-living one (*Naegleria gruberi*). After comprehensively comparing each aspect of the system among these excavates and also with those of archaea and common eukaryotes, we found all the three *Giardia* isolates to harbor a same simplified 5S rRNA system, which is not only much simpler than that of common eukaryotes but also the simplest one among those of these excavates, and is surprisingly very similar to that of archaea; we also found among these excavates the system in parasitic species is not necessarily simpler than that in free-living species, conversely, the system of free-living species is even simpler in some respects than those of parasitic ones.

**Conclusion/Significance:**

The simplicity of *Giardia* 5S rRNA system should be considered a primitive rather than parasitically-degenerated feature. Therefore, *Giardia* 5S rRNA system might be a primitive system that is intermediate between that of archaea and the common eukaryotic model system, and it may reflect the evolutionary history of the eukaryotic 5S rRNA system from the archaeal form. Our results also imply *G. lamblia* might be a primitive eukaryote with secondary parasitically-degenerated features.

## Introduction

5S rRNA is a highly conserved component of large ribosomal subunit in all organisms, excepting only certain mitochondrial systems [1]. In prokaryotes, 5S rRNA is generally transcribed in a common transcription unit with 16S and 23S rRNAs, while in most eukaryotes, 5S rRNA gene is located separately from 18S, 5.8S and 28S rRNA genes and is transcribed alone by RNA polymerase III (RNA pol III) with the participation of a specific transcription factor IIIA (TFIIIA). Here TFIIIA not only acts as an essential transcription factor by binding to the 5S rRNA gene but also combines with 5S rRNA itself to form a 7S ribonucleoprotein (RNP) particle to stabilize the rRNA and facilitate its nuclear export [2], [3]. Ribosomal protein L5 is also known to bind specifically to 5S rRNA and to be involved in its cytoplasmic-nucleolar transport. In eukaryotes, prior to ribosome assembly, the 5S rRNA transcript changes binding partners, trading TFIIIA for L5, and is then escorted to the nucleolus by the latter [4]. In the present work, we refer to 5S rRNA and these associated proteins as the 5S rRNA system. From the above, we find that the components and function of this system are very clear, making it a suitable candidate for investigation across diverse organisms for the purposes of a comparative evolutionary study.

*Giardia lamblia* is one of the most widespread intestinal protozoan parasites in the world. It was once thought to be the most primitive extant eukaryote because it has many so-called primitive molecular and cellular traits [5] and branches out very early from the eukaryote trunk on many phylogenetic trees [6]–[10]. But, actually *Giardia*’s evolutionary position has been under controversy since the proposition of its being extreme primitive eukaryote [11], especially after the discovery of its mitochondrial remnant organelle mitosome [12]. Recently, phylogenomic analyses based on large phylogenomic datasets have placed *G. lamblia* within excavate, one of the three primary divisions of eukaryotes [13]. However, no matter it is primitive or not, it is amazing that it was reported that in this organism no 5S rRNA was detected in either experiments or BLAST searches against its genome database [14], [15], since 5S rRNA is so ubiquitous and highly conserved in both prokaryotic and eukaryotic cells. For a long time, there were no 5S rRNA annotations in the *G. lamblia* genome database before we began the present study. Later, 5S rRNA genes, though there were only eight copies, far fewer than the number seen in most common eukaryotes, were annotated in *G. lamblia* genome database [16]. Studies on some *G. lamblia* 5S rRNA-associated proteins revealed that they are much simpler than those of typical eukaryotes. For example, only one subunit specific to RNA pol III, called C34, was found, and none of the transcriptional factors of RNA polymerase III (TFIIIA, TFIIIB, or TFIIIC) were found either. There were only two components of TFIIIB (TBP and BRF) identified in the *G. lamblia* genome database [17]. But it is unknown whether the entire *G. lamblia* 5S rRNA system is simpler than that of common eukaryotes yet.

Some authors consider the simplicity of many aspects of *G. lamblia*, including genomic reduction, to be the results of parasitic degeneration rather than primitive traits [16], [18]–[20]. We doubt that this is the case for certain aspects of this organism, such as 5S rRNA system, which have no direct relationship with parasitic lifestyle but are rather involved in the fundamental functions of a eukaryotic cell. To confirm this, by collecting published and identifying new data of 5S rRNA system, we compared the *G. lamblia* 5S rRNA system to those of two parasitic species (*Trichomonas vaginalis* and *Trypanosoma cruzi*) and one free-living species (*Naegleria gruberi*), which here served as the representatives with genome database in other three subgroups of excavate, respectively, and also to those of archaea and common eukaryotes. In addition, the genome databases of two other *G. lamblia* isolates, assemblage B isolate GS and assemblage E isolate P15, are now available [21], [22]. In this way, we identified their 5S rRNA system to confirm whether the simplicity of the 5S rRNA system is a common trait among different *G. lamblia* isolates.

## Materials and Methods

### Database

Data regarding genomes and the annotated transcripts and proteins of *G. lamblia* assemblage A isolate WB, B isolate GS, and E isolate P15 were downloaded from the GiardiaDB (http://giardiadb.org/giardiadb/). Genomic data regarding *T. vaginalis* (http://trichdb.org/trichdb/), *T. cruzi* (http://tritrypdb.org/tritrypdb/), and *N. gruberi* (http://genome.jgi-psf.org/Naegr1/Naegr1.home.html) were downloaded from their respective databases.

### Data Collection and Identification

We collected previously reported data on the 5S rRNA system, including the 5S rRNA gene, RNA pol III, associated transcriptional factors, and L5, in *G. lamblia* (including isolates WB, GS, and P15), *T. vaginalis*, *T. cruzi*, and *N. gruberi*. They are summarized as follows: 1) The 5S rRNA gene and its promoters: Eight 5S rRNA genes were reported in the genome database of *G. lamblia* WB and six in the P15 database [16], [22]. The 5S rRNA gene and its TATA box and internal control region (ICR) were reported in *T. vaginalis* [23]. 5S rRNA genes and their ICRs were identified in *T. cruzi* [24]; 2) transcriptional factors and RNA pol III-specific subunits: a TFIIIB with only two components and a RNA pol III with only the specific subunit, C34, were previously reported in *G. lamblia* WB [17]; 3) L5 sequences of *G. lamblia* (including WB, GS, and P15), *T. vaginalis*, *T. cruzi*, and *N. gruberi* were all obtained through the Entrez system at NCBI (http://www.ncbi.nlm.nih.gov/sites/gquery).

The data not already published were identified as follows:

### 5S rRNA Gene

An *in silico* search for the 5S rRNA genes of *G. lamblia* GS and *N. gruberi* was performed using the BLASTn program against the *G. lamblia* GS genome database and *N. gruberi* genome database [21], [25]. The 5S rRNA gene sequences from prokaryotes and eukaryotes that were used as query sequences were retrieved from the 5S rRNA database (http://www.man.poznan.pl/5SData) [26]. BLAST search default parameters were modified to reduce stringency: the penalty for a gap or a mismatch was changed from 3 to 1 to enable us to identify the 5S rRNA gene. The 5S rRNA genes of *G. lamblia* WB and P15 were used as queries to search for 5S rRNA gene against the *G. lamblia* GS genome database by using the BLAST program (http://giardiadb.org/giardiadb/).

Data regarding the internal control region (ICR) and TATA box were found in the *G. lamblia* 5S rRNA genes in the following manner: For ICR, the consensus sequences of its three elements: Box A [5′-N(G/C)(C/T)(C/T)AANCNNNNNNN-3′], intermediate element (IE) [5′-(C/G)NN(G/A)(G/A)N-3′], and Box C [5′-NNG(G/A)TGGGNG(T/A)CCN(C/T)NNG-3′] were searched manually within the *G. lambia* 5S rRNA genes. For the TATA box, its highly conserved core sequence (5′-TATAAA-3′) was used as a consensus sequences to analyze the upstream sequences of *G. lamblia* 5S rRNA genes. Because of the flexible position of the TATA box, an AT-rich region, being relative to the transcriptional start site in *G. lamblia*, the entire 60 bp upstream region of the transcriptional start site of *G. lamblia* 5S rRNA genes were analyzed. Using these same methods, the ICR of 5S rRNA gene in *N. gruberi* and TATA boxes in the 5S rRNA genes of *T. cruzi* and *N. gruberi* were identified.

### TFIIIA

No TFIIIA had previously been identified in *G. lamblia* WB by homolog search against its partial genome assemblies [17]. Once the fully sequenced *G. lamblia* WB genome database was available, we tried to identify TFIIIA via BLASTp and PSI-BLAST searches against the database again. Considering the high primary sequence divergences of TFIIIA among different species, we used the C2H2 motif of TFIIIA as query. The C2H2 motif-containing sequences were obtained from the PROSITE database (release 32 of the PROSITE pattern ZINC-FINGER_C2H2, Accession No. PS00028). Then the best hits were filtered by the presence of C2H2 zinc finger domains using the Interproscan program [27]. TFIIIA was identified in *G. lamblia* GS and P15, *T. vaginalis*, *T. cruzi*, and *N. gruberi* by BLASTp and PSI-BLAST searching against their corresponding genome databases.

### TFIIIB, TFIIIC, and Specific Subunits of RNA Pol III

The data regarding TFIIIB, TFIIIC and specific subunits of RNA pol III in *G. lamblia* were reported previously by BLAST searching against the *G. lamblia* WB partial genome assembly [17]. In this work, we again found them in the fully sequenced genome database. *Saccharomyces cerevisiae* sequences of subunits of TFIIIB and TFIIIC and of the specific subunits of RNA pol III were used as queries to BLASTp search against genome databases of all the three *G. lamblia* isolates, *T. vaginalis*, *T. cruzi*, and *N. gruberi*, to get putative homologs. Then the putative homologs were assessed using domain information in the Pfam database (http://pfam.sanger.ac.uk) [28]. Another BLASTp search was performed, this time against the non-redundant (nr) NCBI protein database. Sequences lacking characteristic domains of the given proteins or indicating other proteins were discarded. For proteins for which no homologs could be found by BLASTp search, at least five iterations of PSI-BLAST search was carried out online to identify putative homologs. Then these putative homologs were confirmed by Pfam domain analysis and BLASTp against the NCBI nr database, as described above.

### RT-PCR and Sequencing

To examine the transcription activities of the *G. lamblia* 5S rRNA genes, reverse transcription polymerase chain reaction (RT-PCR) experiments were carried out as follows: Small RNAs (≤200 nucleotides) were extracted from *G. lamblia* WB cells using a mirVana™ miRNA Isolation Kit (Ambion, Austin, TX, U.S.) according to the manufacturer’s instructions. Then the small RNAs were polyadenylated at 37°C for 30 min in 60 µl of reaction mixture with 500 ng small RNAs, 3 mM rATP, 6 µl 10×Buffer and 15 U *E. coli* poly(A) polymerase (New England BioLab), and the poly(A)-tailed RNAs were recovered by phenol/chloroform extraction and ethanol precipitation. Finally the purified RNAs were used as templates for RT-PCR, using a RNA PCR Kit (TaKaRa Biotechnology Co., Ltd., Dalian, China.) according to the manufacturer’s instructions. The gene-specific primers were designed according to the sequence of our identified *G. lamblia* 5S rRNA genes. Primers ((Pfr 1∶5′-TCggCCATCCTACggCggAAACT-3′; Pfr 2∶5′-CCCACgACgTCTCCgATCgCAgT-3′) were synthesized by Sangon (Sangon Ltd., Shanghai, China).

The purified products of these RT-PCR experiments were cloned into PMD 19-T vectors (TaKaRa). Twenty clones were selected at random and sequenced at Sangon Ltd.

## Results

### Identification of 5S rRNA Genes in *G. lamblia* (WB, GS, and P15) and *N. gruberi*

Our BLASTn search included modified parameters to reduce stringency. It confirmed that there are only eight 5S rRNA genes located separately in the *G. lamblia* WB database and six in the P15 database. This is consistent with the annotations in these two databases [16], [22]. Based on the genome sequence, seven of the eight *G. lamblia* WB 5S rRNA genes each were found to have a putative 5S rRNA coding region 117 bp in length while the other had an extra 15 bp insert within the coding region. However, in our RT-PCR and sequencing experiments, the products detected were only from the 117 bp genes. Thus, the gene with the extra insert might not be transcribed.

**Figure 1:**
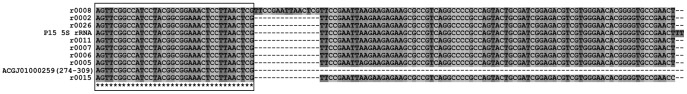
Sequence alignments of 5S rRNA genes of the three *G. lamblia* isolates. r0002, r0005, r0006, r0007, r0011, r0015, r0026, and r0008 represent the eight *G. lamblia* WB 5S rRNA genes. ACGJ01000259 (274–309) represents sequences from residue 274 to 309 of contig ACGJ01000259 in the *G. lamblia* GS genome. Box denotes the putative partial 5S rRNA gene identified in *G. lamblia* GS sharing 100% identity with 5′ fragment of 5S rRNA gene in *G. lamblia* WB and P15.

No putative 5S rRNA gene was identified in the *G. lamblia* GS genome database upon the retrieval of various 5S rRNA sequences from the 5S rRNA database as queries to BLASTn search against the genome database. However, one hit (Contig ID: ACGJ01000259), which was found to possess a 36 bp sequence sharing 100% identity with the 5′ terminal region (from+1 bp to 36 bp) of 5S rRNA genes of *G. lamblia* WB and P15 was found when the 5S rRNA genes of the two isolates were used as queries (Figure 1). The region in contig ACGJ01000259 (309 bp long) sharing 100% identity with 5S rRNA gene (from+1 bp to 36 bp) of *G. lamblia* WB and P15 was found at the end of this contig (from 274 bp to 309 bp). Moreover, a 79 bp RNA fragment sharing 99% identity with the 3′ terminal region (from +39 bp to +117 bp) of *G. lamblia* WB 5S rRNA was once reported in *G. lamblia* GS [21]. In this way, the lack of a *G. lamblia* 5S rRNA gene in the *G. lamblia* GS genome database is most probably due to the numerous gaps in the database and the small number genes in this organism.

Using the BLASTn program to search against the *N. gruberi* genome database, we found 279 potential 5S rRNA genes (Table S1).

### Comparisons of 5S rRNA Genes among *G. lamblia*, and Other Three Excavates, Common Eukaryotes, and Archaea

According to the above identified 5S rRNA genes in *G. lamblia* and *N. gruberi*, and the reported many copies of 5S rRNA gene in *T. vaginalis* and *T. cruzi* [23], [24], it can be said that in contrast to common eukaryotes and the other three investigated excavates, *G. lamblia* had a very small copy number of 5S rRNA genes and no tandem 5S rRNA gene regions, suggesting that it is very similar to archaea (Table 1).

**Table 1:** Comparisons of 5S rRNA system among four representatives of excavate and with archaea and common eukaryotes.

Organism			Archaea	Excavate						Common eukaryotes
				*Gl*			*Tv*	*Tc*	*Ng*	
				WB	GS	P15				
5S rRNA gene	Copy number		Few [^48^]	8 [^16^]	Y* (?)	6 [^21^]	Many [^23^]	≈1600 [^24^]	279*	Many
	Tandem arrangement		N	N	N*	N	Y	Y	Y*	Y
	Located with rDNA Units		Y	N	N*	N	N	N	N*	N
Promoter	ICR		N	N*	N*	N*	Y [^23^]	Y [^24^]	Y*	Y
	TATA-box		Y	Y*	N*	N*	Y [^23^]	N*	Y*	Y
Transcription factor	TFIIIA		N	N	N*	N*	Y*	N*	N*	Y
	TFIIIB	TBP	Y [^40^]	Y [^17^]	Y*	Y*	Y*	Y*	Y*	Y
		TFB/BRF	Y [^40^]	Y[^17^]	Y*	Y*	Y*	Y*	Y*	Y
		B′′	N	N	N*	N*	Y*	Y*	Y*	Y
	TFIIIC	TFIIIC 102	N	N	N*	N*	Y*	Y*	Y*	Y
		TFIIIC 63	N	N	N*	N*	Y*	Y*	Y*	Y
RNA pol III specific subunit	C34		Y [^17^]	Y [^17^]	Y*	Y*	Y*	Y*	Y*	Y
	C82		N	Y*	Y*	Y*	Y*	N*	Y*	Y
	C31		N	N	N*	N*	N*	N*	N*	Y
	C17		N	N	N*	N*	N*	N*	Y*	Y
	C53		N	N	N*	N*	Y*	Y*	Y*	Y
L5 protein			Y [^46^]	Y	Y	Y	Y	Y	Y	Y

Note: Y, present; N, absent; (?), unknown copy number;

* identified in this work.

Organism abbreviations: *Gl: Giardia lamblia*; *Tv: Trichomonas vaginalis*; *Tc: Trypanosoma cruzi*; *Ng: Naegleria gruberi*.

**Figure 2:**
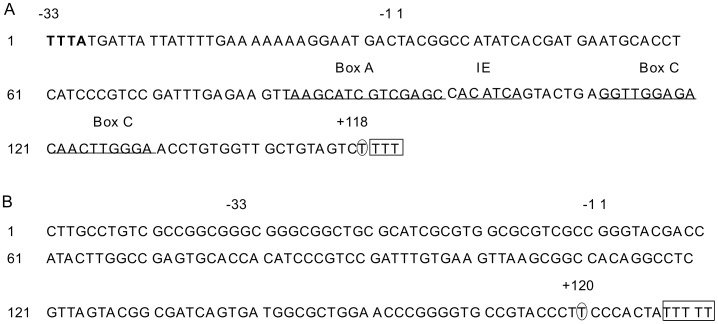
5S rRNA gene and its upstream sequences of *N. gruberi* and *T. cruzi*. A ) The putative ICR promoter elements identified in *N. gruberi* 5S rRNA gene. The TATA box is denoted in bold. Underlined sequences refer to the putative ICR. The proposed first transcribed base (T) is positioned with 1 and the putative last nucleotide of the mature molecule is shown circled (T in position +118). Empty boxes indicate T-runs as putative termination sequences. **B**) No TATA box could be identified in the *T. cruzi* 5S rRNA gene.

Analysis on putative promoter elements revealed that there are no special relevance to the intermediate element [IE: 5′-(C/G)NN(G/A)(G/A)N-3′], the Box A [5′-N(G/C)(C/T)(C/T)AANCNNNNNNN-3′], or Box C [5′-NNG(G/A)TGGGNG(T/A)CCN(C/T)NNG-3′] of the ICR in any part of the *G. lamblia* WB or P15 5S rRNA genes. This indicates that these genes do not possess ICR and would not bind TFIIIA specifically during transcription, as found in common eukaryotes. Our analysis showed that presence of ICR in the 5S rRNA genes of *N. gruberi* (Figure 2). The presences of ICR have been reported previously in the 5S rRNA genes of *T. cruzi* and *T. vaginalis* [23], [24]. This suggests that the three other excavates, both parasitic and free-living, are similar to common eukaryotes in this respect.

**Figure 3:**
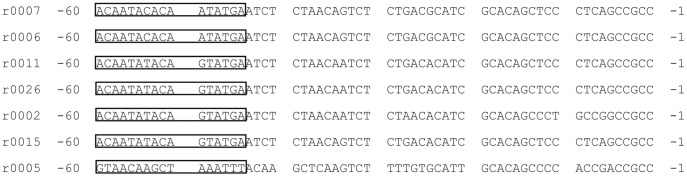
TATA-like box identified in *G. lamblia* WB 5S rRNA gene. The regions between −58 and −43 bp upstream the transcriptional initiation site of *G. lamblia* WB 5S rRNA genes all contain an AT-rich region (denoted by box), which might be the TATA-like box of *G. lamblia* WB 5S rRNA gene.

However, although no canonical TATA box was found in *G. lamblia* 5S rRNA genes, we have identified a 16 bp AT-rich region between −58 and −43 bp upstream of the transcriptional initiation site of these genes, whose consensus sequence is 5′-AA(T/C)A(C/T/A)(A/G) C(A/T)(G/A)(T/A)AT(G/T)(T/A)A(T/C)-3′ (Figure 3). Previous studies have indicated that the TATA boxes of *G. lamblia* genes are not as canonical as those of common eukaryotes. Rather, they vary with respect to their sequence, length, and exact position relative to the transcription start site [29]–[33]. Our analysis also showed a TATA box in the 5S rRNA genes of *N. gruberi* but not in *T. cruzi* (Figure 2). *T. vaginalis* has been reported to have TATA box in its 5S rRNA genes [23] (Table 1).

### Identification and Comparison of Transcriptional Factors and Specific Subunits of RNA Pol III

TFIIIA was found in *T. vaginalis* but not in *T. cruzi*, *N. gruberi,* or the three *G. lamblia* isolates upon BLASTp or PSI-BLAST search against their corresponding genome databases. We then used a C2H2 motif as a query for another BLASTp search against the *G. lamblia* WB genome database to confirm whether TFIIIA is really absent from it. After the BLASTp search, we found 1,959 best hits. Of these, 102 were predicted to be putative zinc finger proteins, and 18 out of these 102 proteins were further predicted to be C2H2-type zinc finger proteins. But 17 out of the 18 proteins each contained only 1–2 zinc finger repeats, and the other one (ORF: 17003) had a total of four repeats. In common eukaryotes, the number of TFIIIA finger repeats is usually nine. None of the 18 C2H2-type zinc finger proteins were annotated as TFIIIA in the NCBI database (Table S2).

**Figure 4:**
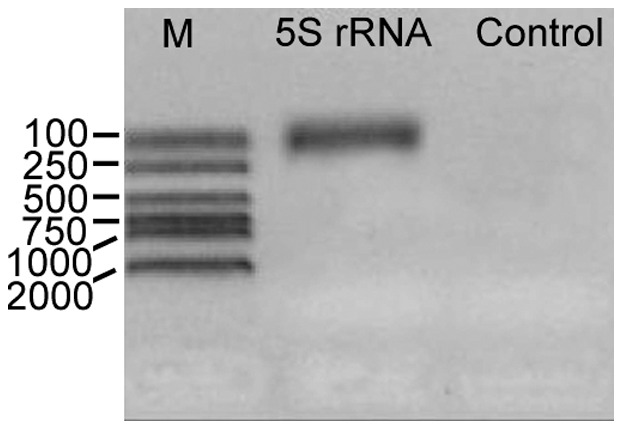
Results of the RT-PCR experiment of the identified *G. lamblia* WB 5S rRNA gene. M, marker.

Our investigation further confirmed that this organism lacks TFIIIB and TFIIIC, but two components of TFIIIB were present in *G. lamblia* WB as previously reported. In *G. lamblia* GS and P15, similarly, no TFIIIB or TFIIIC was found, but two components of TFIIIB were identified. However, both TFIIIB and TFIIIC were found in all the other three investigated excavates, *T. vaginalis*, *T. cruzi*, and *N. gruberi* (Table S3).

Of the five specific subunits of RNA pol III (C17, C31, C34, C53, and C82), two (C34 and C82) were identified in *G. lamblia* WB, GS, and P15 by our PSI-BLAST. Of these, only one, C34, had been reported in *G. lamblia* WB before this work [17]. Two, C34 and C53, were found in *T. cruzi*; three, C34, C53, and C82, were identified in *T. vaginalis*; and four, C34, C53, C82, and C17, were found in the free-living species *N. gruberi* (Table S3).

### Examination of Transcription of *G. lamblia* 5S rRNA Gene

Our RT-PCR experiment on the *G. lamblia* WB 5S rRNA genes showed positive results (Figure 4). The sequencing results indicated the RT-PCR products were the exact transcripts of the seven *G. lamblia* WB 5S rRNA genes with the normal length (117 bp), while no transcript corresponds to the other one which has a 15 bp insertion. Therefore, the seven normal-length *G. lamblia* WB 5S rRNA genes were found to be actively transcribed while the other was not.

## Discussion

By collecting and confirming the existing data and identifying new data, we were able to construct almost complete datasets of the 5S rRNA systems of the three sequenced isolates of *G. lamblia* and three other excavates. Therefore, in this study, we were able to comprehensively compare each aspect of the 5S rRNA system among them and with those of archaea and common eukaryotes.

In the genomes of common eukaryotes, 5S rRNA genes are usually arranged in tandem arrays of several hundreds and thousands of copies in several different rDNA regions. The total gene copy number varies widely by species. Humans have 1700–2000 copies and *Xenopus* oocytes have ≈20,000 [34], [35]. Excavates, *Crithidia fasciculata* have ≈250, *T. vaginalis* and *T. cruzi* each have hundreds and thousands of copies [23], [24], [36]. There are 279 putative 5S rRNA genes in *N. gruberi*. However, our study has indicated that in all the three sequenced *G. lamblia* isolates, only a few copies of 5S rRNA genes exist. The total gene copy number in *G. lamblia* is unexpectedly small, and the genes are not arranged in tandem. This is not consistent with the pattern observed in common eukaryotes and in other excavates (both free-living and parasitic) but rather with those in archaea. On the other hand, the *G. lamblia* 5S rRNA genes are located far from the 18S-5.8S-28S rRNA gene unit, as found in common eukaryotes, suggesting they are also transcribed independently by RNA pol III.

In this study we found that there are no ICR but rather a gene-external TATA-like box in each *G. lamblia* 5S rRNA gene. Most eukaryotes have both [37]. In the other three excavates studied, both well defined gene-external TATA box and gene-internal ICR appear in *T. vaginalis* 5S rRNA genes [23], and both were also found in 5S rRNA genes of *N. gruberi* in the present study, however, there is only gene-internal ICR in *T. cruzi* 5S rRNA genes [24]. The characteristics of 5S rRNA gene promoters in these excavates suggests which kind of promoter appears in a species has nothing to do with its lifestyle. However, the results of our study indicate that the *G. lamblia* 5S rRNA gene promoter might be a very primitive, and similar to that of archaea. Moreover, this might further supports the idea that during evolution of eukaryotes, the gene-external TATA box promoter of the 5S rRNA genes might have been inherited from archaea and emerge very early in the evolution of eukaryotes, while the gene-internal ICR arose at later point after the divergence of *G. lamblia* from the eukaryotic trunk.

Usually, three transcription factors are involved in the transcription of 5S rRNA by RNA pol III in common eukaryotes, and TFIIIA is the only one specific to this process. The binding of TFIIIA to the ICR of 5S rRNA gene is the first step of the transcription of this gene. This allows the recruitment of TFIIIC, TFIIIB, and RNA pol III [38]. TFIIIA also combines with 5S rRNA to export it from the nucleus to the cytoplasm. Up to now, knowledge about TFIIIA has mainly come from studies of higher eukaryotes, and no TFIIIA has ever been reported in protists. One previous study of *G. lamblia* reported finding no TFIIIA [17]. Considering that this previous study was based on partial *G. lamblia* genome assemblies, the present study used the complete *G. lamblia* genome databases and relatively conserved C2H2 domain of TFIIIA as query to search TFIIIA, but still identify no homolog to the typical eukaryotic TFIIIA in the three isolates of *G. lamblia*. Our sequence analysis also revealed that the absence of ICR in the *G. lamblia* 5S rRNA gene, which probably also implies the absence of TFIIIA. These findings further confirm the absence of TFIIIA in *Giardia*, and suggest that *G. lamblia* 5S rRNA transcription is TFIIIA-independent. Previously, TFIIIA-independent 5S rRNA transcription in eukaryotes was only reported in *Yarrowia lipolytica*, where 5S rRNA gene is co-transcribed with tRNA [39]. But no *G. lamblia* 5S rRNA genes have a neighboring location with tRNA gene as in *Y. lipolytica* according to our investigation. Furthermore, there is no homologs of TFIIIB and TFIIIC in *G. lamblia* [17], which was also further confirmed in the present study. These findings may indicate that the initiation mechanism of *G. lamblia* 5S rRNA transcription is different from those of all eukaryotes studied. Although it does not have TFIIIB, *G. lamblia* possesses two important subunits of TFIIIB: TATA box binding protein (TBP) and TFIIB-related factor (BRF) [17]. In archaea, TBP and TFB can mediate the initiation of basal transcription [40]. Archaeal TFB and eukaryotic BRF are evolutionarily related by sharing a common ancestry [17]. For this reason, the initiation mechanism of *G. lamblia* 5S rRNA transcription should be similar to that of archaea, specifically, with respect to without the participation of TFIIIA, TFIIIB, and TFIIIC but including TBP and TFB/BRF. As for the other three investigated excavates, both TFIIIB and TFIIIC exist in all of them, while TFIIIA only exists in the parasitic excavate *T. vaginalis* and not in the free-living *N. gruberi*. This means that parasitism does not necessarily lead to the reduction of the transcription factors of RNA pol III. Therefore, the simplicity of the *G. lamblia* transcription factor system should not be interpreted as necessarily the results of parasitic degeneration but most probably as a primitive feature of this organism.

The three eukaryotic RNA polymerases (pol I, II, III) all have both common subunits and subunits specific to each of themselves, whereas *G. lamblia* RNA pol III has been reported to possess all the common subunits but lack all specific subunits except for C34 [17]. However, besides C34, we found the fully sequenced *G. lamblia* genome to include another specific subunit, C82. In yeast, subunit C34 was shown to interact with subunits C82 and C31 to form a subcomplex functioning in the initiation of RNA pol III transcription [41]. Subunit C34 and C31 were shown to play a key role in determining the initiation specificity of RNA pol III transcription [42], [43]. Archaeal RNA polymerase has recently been reported to have one ortholog to C34, which might be involved in the transcription of non-coding RNAs [44]. Our investigation revealed the presence of C34 and C82 in all the investigated excavates (except *T. cruzi*, which lacked C82, probably due to secondary loss), while C31 was absent from all of them. These mean that C34 might have been inherited from archaea and emerged very early in the evolution of eukaryotes, while C31 arose at least after the divergence of excavate from the eukaryotic trunk. Lacking C31, the RNA pol III of excavate might carry out the specific initiation of RNA pol III transcription using only C34 and C82. This is similar to the initiation of non-coding RNA transcription only with C34 in archaea. However, the parasitic excavates *T. vaginalis* and *T. cruzi* also have another more specific subunit that is absent from *G. lamblia*, C53. Therefore, the similarity of *G. lamblia* RNA pol III to those of archaea also seem not to be due to parasitic degeneration but to be the very primitive traits of this organism.

Since TFIIIA also functions as a 5S rRNA transcript chaperone required for the nuclear-cytoplasmic transport in common eukaryotes [3], [45], without TFIIIA, the 5S rRNA nuclear-cytoplasmic transport in *G. lamblia* must be unique. Whereas, the presence of 5S rRNA-associated protein L5 in *G. lamblia*, however, suggests that the mechanisms of *G. lamblia* 5S rRNA cytoplasmic-nucleolar transport and incorporation into large ribosomal subunit are similar to that of common eukaryotes. No TFIIIA has yet been reported in archaea, but L5 protein has been reported [46], [47]. The *G. lamblia* 5S rRNA transport system can then be said to be similar to that in archaea. The fact that TFIIIA is present in the parasitic excavate *T. vaginalis* but absent from the free-living *N. gruberi* further suggests that the absence of TFIIIA in *G. lamblia* is not necessarily due to parasitic degeneration but most probably to the primitive nature of this organism.

In summary, our results reveal a unique 5S rRNA system in *G. lamblia*: On one hand, some of its features, such as the separated location of the 5S rRNA genes and the transcription of 5S rRNA genes by RNA pol III, are consistent with that seen in common eukaryotes. On the other hand, many other traits, such as the lack of a tandem 5S rRNA gene region, the low gene copy number, the relatively simple transcription system (lacking the participation of TFIIIA, TFIIIB, and TFIIIC but inclusion of TBP, BRF, the RNA pol III with only two specific subunits, C34 and C82), and the TFIIIA-independent but L5-dependent 5S rRNA transport and incorporation system, are much simpler than those of most eukaryotes and very similar to those of archaea. Comparisons of the entire 5S rRNA system among the three sequenced *G. lamblia* isolates and with the other three investigated excavates showed 1) All the three *G. lamblia* isolates harbor the same simplified 5S rRNA system, which is the simplest one among all the investigated excavates in almost every aspect of the system; 2) parasitic species are not definitely simpler than that of free-living ones, conversely, the free-living species can be simpler in some reaspects of the system, such as transcription factors (Table 1). In fact, as an obligate parasite, though not an excavate, *Encephalitozoon cuniculi* was also compared to *G. lamblia* by us. We found the 5S rRNA system of *G. lamblia* is to be simpler than that of *E. cuniculi*, especially in the aspect of transcription factors (Table S4). In this way, the simplicity of *G. lamblia* 5S rRNA system should not be considered due to parasitic degeneration. If this is not ture, then it would become difficult to explain why the system was definitely degenerated to be an archaeal form.

In conclusion, *G. lamblia* represents an ancient primitive eukaryotic 5S rRNA system intermediate to archaea and the common eukaryotic model system. It may reflect the history of many aspects of the typical eukaryotic 5S rRNA system as it evolved from its archaeal form. Simultaneously, our work implies although the adaptation to a parasitic lifestyle has caused a number of profound changes to *G. lamblia*, such as a compact genome, mitochondrial relics, and simplified machinery for some metabolic pathways, some very primitive eukaryotic features have been retained by this organism. These have no direct relationship with the parasitic lifestyle but are rather involved in the fundamental functions of a eukaryotic cell. Therefore, we propose that *G. lamblia* is most likely a combination of eukaryotic primitiveness and secondary parasitic degeneration.

## Supporting Information

Table S1 **Putative 5S rRNA genes identified in N. gruberi genome database.**
(DOC)

Table S2 **The identified C2H2-type zinc finger proteins in G. lamblia WB genome.**
(DOC)

Table S3 **Accession numbers of genes of transcriptional factors and specific subunits of RNA pol III in G. lamblia, T. vaginalis, T. cruzi, and N. gruberi.**
(DOC)

Table S4 **Comparisons of the 5S rRNA system between G. lamblia and Encephalitozoon cuniculi.**
(DOC)
