# Supplementary material for: Comparative Analysis of the 5S rRNA and Its Associated Proteins Reveals Unique Primitive Rather Than Parasitic Features in Giardia lamblia
Source: PLoS One. 2012 Jun 7;7(6):e36878. doi: 10.1371/journal.pone.0036878 (PMC3369914; doi:10.1371/journal.pone.0036878)
Supplement: Table S1 — Putative 5S rRNA genes identified in N. gruberi genome database. (DOC) [file pone.0036878.s001.doc]

**Table S1. Putative 5S rRNA genes identified in *N. gruberi* genome database.**

| **RNA_ID** | **Scaffold** | **Start** | **End** | **Similarity** |
| --- | --- | --- | --- | --- |
| R32613 | scaffold_59 | 172675 | 172754 | 0.5 |
| R54191 | scaffold_42 | 267547 | 267634 | 0.521008403 |
| R32519 | scaffold_61 | 144509 | 144592 | 0.533898305 |
| R33922 | scaffold_1084 | 570 | 653 | 0.541666667 |
| R20702 | scaffold_594 | 1 | 86 | 0.541666667 |
| R33827 | scaffold_19 | 112737 | 112821 | 0.55 |
| R32403 | scaffold_27 | 25164 | 25250 | 0.576271186 |
| R32813 | scaffold_61 | 101584 | 101671 | 0.593220339 |
| R33886 | scaffold_6 | 271664 | 271762 | 0.633333333 |
| R32819 | scaffold_73 | 113252 | 113351 | 0.644067797 |
| R54247 | scaffold_4 | 642712 | 642814 | 0.647058824 |
| R32615 | scaffold_58 | 6842 | 6947 | 0.652542373 |
| R62983 | scaffold_35 | 85175 | 85277 | 0.655462185 |
| R32335 | scaffold_54 | 275852 | 275950 | 0.669491525 |
| R32334 | scaffold_54 | 144229 | 144326 | 0.669491525 |
| R33567 | scaffold_11 | 502793 | 502904 | 0.7 |
| R33370 | scaffold_41 | 303199 | 303315 | 0.708333333 |
| R9415 | scaffold_935 | 806 | 920 | 0.719008264 |
| R33912 | scaffold_81 | 52665 | 52781 | 0.725 |
| R33534 | scaffold_20 | 227488 | 227604 | 0.725 |
| R33822 | scaffold_20 | 132468 | 132584 | 0.725 |
| R33885 | scaffold_6 | 324854 | 324970 | 0.725 |
| R33898 | scaffold_2 | 75812 | 75928 | 0.725 |
| R33878 | scaffold_7 | 543593 | 543709 | 0.725 |
| R33448 | scaffold_56 | 231625 | 231741 | 0.725 |
| R9404 | scaffold_879 | 925 | 1043 | 0.727272727 |
| R32243 | scaffold_43 | 164721 | 164832 | 0.728813559 |
| R32769 | scaffold_7 | 187831 | 187942 | 0.728813559 |
| R33419 | scaffold_85 | 152605 | 152721 | 0.733333333 |
| R33609 | scaffold_3 | 545090 | 545206 | 0.733333333 |
| R33360 | scaffold_76 | 43677 | 43793 | 0.733333333 |
| R33913 | scaffold_39 | 296936 | 297052 | 0.733333333 |
| R33911 | scaffold_81 | 50274 | 50390 | 0.733333333 |
| R33667 | scaffold_12 | 420559 | 420675 | 0.733333333 |
| R33897 | scaffold_2 | 111262 | 111378 | 0.733333333 |
| R33384 | scaffold_12 | 422722 | 422838 | 0.733333333 |
| R33754 | scaffold_50 | 496365 | 496480 | 0.733333333 |
| R33463 | scaffold_52 | 212551 | 212667 | 0.733333333 |
| R33737 | scaffold_55 | 169752 | 169868 | 0.733333333 |
| R33656 | scaffold_41 | 145487 | 145603 | 0.733333333 |
| R33875 | scaffold_7 | 545993 | 546109 | 0.733333333 |
| R33599 | scaffold_6 | 466429 | 466545 | 0.733333333 |
| R33462 | scaffold_52 | 57620 | 57736 | 0.733333333 |
| R33792 | scaffold_31 | 173492 | 173608 | 0.733333333 |
| R33731 | scaffold_56 | 42219 | 42335 | 0.733333333 |
| R33363 | scaffold_43 | 168060 | 168176 | 0.733333333 |
| R33799 | scaffold_29 | 307268 | 307384 | 0.733333333 |
| R33755 | scaffold_50 | 344821 | 344937 | 0.733333333 |
| R33909 | scaffold_1546 | 561 | 677 | 0.733333333 |
| R35561 | scaffold_636 | 2060 | 2179 | 0.733333333 |
| R33762 | scaffold_47 | 244821 | 244937 | 0.733333333 |
| R33767 | scaffold_45 | 179650 | 179766 | 0.733333333 |
| R33704 | scaffold_85 | 163606 | 163722 | 0.733333333 |
| R33821 | scaffold_20 | 144093 | 144209 | 0.733333333 |
| R33910 | scaffold_108 | 14486 | 14602 | 0.733333333 |
| R9397 | scaffold_158 | 11537 | 11655 | 0.73553719 |
| R22828 | scaffold_7 | 384142 | 384259 | 0.73553719 |
| R21410 | scaffold_33 | 70414 | 70530 | 0.73553719 |
| R33452 | scaffold_55 | 254742 | 254858 | 0.741666667 |
| R35530 | scaffold_7 | 578765 | 578884 | 0.741666667 |
| R33622 | scaffold_74 | 7102 | 7218 | 0.741666667 |
| R33882 | scaffold_6 | 112307 | 112423 | 0.741666667 |
| R33865 | scaffold_9 | 605171 | 605287 | 0.741666667 |
| R33899 | scaffold_816 | 817 | 933 | 0.741666667 |
| R33905 | scaffold_87 | 125774 | 125890 | 0.741666667 |
| R33728 | scaffold_57 | 64763 | 64879 | 0.741666667 |
| R33873 | scaffold_7 | 193327 | 193443 | 0.741666667 |
| R33900 | scaffold_733 | 1434 | 1550 | 0.741666667 |
| R33597 | scaffold_6 | 244672 | 244788 | 0.741666667 |
| R33848 | scaffold_14 | 211111 | 211227 | 0.741666667 |
| R33508 | scaffold_31 | 149302 | 149418 | 0.741666667 |
| R33416 | scaffold_92 | 30911 | 31028 | 0.741666667 |
| R33890 | scaffold_4 | 152053 | 152169 | 0.741666667 |
| R33855 | scaffold_11 | 441256 | 441372 | 0.741666667 |
| R33904 | scaffold_147 | 12196 | 12312 | 0.741666667 |
| R33863 | scaffold_9 | 151876 | 151992 | 0.741666667 |
| R33793 | scaffold_31 | 247674 | 247813 | 0.741666667 |
| R33466 | scaffold_51 | 194458 | 194574 | 0.741666667 |
| R33719 | scaffold_65 | 141241 | 141357 | 0.741666667 |
| R33587 | scaffold_7 | 146362 | 146478 | 0.741666667 |
| R33714 | scaffold_68 | 92738 | 92854 | 0.741666667 |
| R33617 | scaffold_204 | 766 | 882 | 0.741666667 |
| R33917 | scaffold_658 | 147 | 262 | 0.741666667 |
| R33489 | scaffold_42 | 95304 | 95420 | 0.741666667 |
| R33469 | scaffold_50 | 135938 | 136054 | 0.741666667 |
| R33907 | scaffold_74 | 72041 | 72157 | 0.741666667 |
| R33770 | scaffold_44 | 103868 | 103984 | 0.741666667 |
| R33864 | scaffold_9 | 430630 | 430746 | 0.741666667 |
| R35366 | scaffold_76 | 56333 | 56452 | 0.741666667 |
| R33807 | scaffold_27 | 245008 | 245124 | 0.741666667 |
| R33526 | scaffold_24 | 204374 | 204489 | 0.741666667 |
| R33620 | scaffold_84 | 130736 | 130852 | 0.741666667 |
| R33586 | scaffold_7 | 144862 | 144978 | 0.741666667 |
| R33502 | scaffold_33 | 36469 | 36585 | 0.741666667 |
| R33745 | scaffold_52 | 209738 | 209854 | 0.741666667 |
| R33758 | scaffold_49 | 172250 | 172366 | 0.741666667 |
| R33697 | scaffold_93 | 930 | 1046 | 0.741666667 |
| R33373 | scaffold_28 | 233021 | 233137 | 0.741666667 |
| R22614 | scaffold_378 | 6624 | 6741 | 0.743801653 |
| R22632 | scaffold_10 | 271052 | 271169 | 0.743801653 |
| R21271 | scaffold_23 | 152238 | 152355 | 0.743801653 |
| R32435 | scaffold_16 | 438985 | 439096 | 0.745762712 |
| R32729 | scaffold_16 | 449091 | 449202 | 0.745762712 |
| R33400 | scaffold_215 | 110 | 226 | 0.75 |
| R33418 | scaffold_85 | 53800 | 53916 | 0.75 |
| R33675 | scaffold_1194 | 88 | 204 | 0.75 |
| R33421 | scaffold_83 | 18784 | 18900 | 0.75 |
| R33692 | scaffold_166 | 532 | 648 | 0.75 |
| R33585 | scaffold_7 | 551533 | 551649 | 0.75 |
| R33517 | scaffold_27 | 84376 | 84492 | 0.75 |
| R33554 | scaffold_14 | 719563 | 719679 | 0.75 |
| R33665 | scaffold_12 | 35006 | 35122 | 0.75 |
| R33528 | scaffold_21 | 23092 | 23208 | 0.75 |
| R33690 | scaffold_197 | 9071 | 9187 | 0.75 |
| R33388 | scaffold_1200 | 1128 | 1244 | 0.75 |
| R33781 | scaffold_35 | 123355 | 123471 | 0.75 |
| R35318 | scaffold_4 | 100976 | 101095 | 0.75 |
| R33789 | scaffold_31 | 182181 | 182297 | 0.75 |
| R33695 | scaffold_97 | 33426 | 33542 | 0.75 |
| R33893 | scaffold_3 | 540357 | 540473 | 0.75 |
| R33440 | scaffold_59 | 125316 | 125432 | 0.75 |
| R33726 | scaffold_58 | 73271 | 73387 | 0.75 |
| R33834 | scaffold_18 | 436682 | 436798 | 0.75 |
| R33718 | scaffold_65 | 130419 | 130535 | 0.75 |
| R33859 | scaffold_9 | 239784 | 239900 | 0.75 |
| R33851 | scaffold_13 | 175088 | 175204 | 0.75 |
| R33417 | scaffold_85 | 56154 | 56270 | 0.75 |
| R33713 | scaffold_68 | 12147 | 12263 | 0.75 |
| R33484 | scaffold_44 | 132448 | 132564 | 0.75 |
| R33539 | scaffold_18 | 42664 | 42780 | 0.75 |
| R33514 | scaffold_29 | 437459 | 437575 | 0.75 |
| R33752 | scaffold_50 | 145045 | 145161 | 0.75 |
| R33473 | scaffold_49 | 245966 | 246082 | 0.75 |
| R33836 | scaffold_17 | 464811 | 464927 | 0.75 |
| R33493 | scaffold_37 | 359601 | 359717 | 0.75 |
| R33815 | scaffold_21 | 92029 | 92145 | 0.75 |
| R33396 | scaffold_382 | 6695 | 6811 | 0.75 |
| R33734 | scaffold_55 | 252459 | 252575 | 0.75 |
| R33529 | scaffold_20 | 78511 | 78627 | 0.75 |
| R33555 | scaffold_14 | 85321 | 85437 | 0.75 |
| R33439 | scaffold_60 | 148589 | 148705 | 0.75 |
| R33707 | scaffold_79 | 146279 | 146395 | 0.75 |
| R33683 | scaffold_269 | 7750 | 7866 | 0.75 |
| R33850 | scaffold_13 | 177056 | 177172 | 0.75 |
| R33482 | scaffold_45 | 328831 | 328947 | 0.75 |
| R33811 | scaffold_25 | 206512 | 206628 | 0.75 |
| R33427 | scaffold_72 | 167233 | 167349 | 0.75 |
| R33540 | scaffold_18 | 65414 | 65530 | 0.75 |
| R33425 | scaffold_78 | 186496 | 186612 | 0.75 |
| R33386 | scaffold_1658 | 707 | 823 | 0.75 |
| R33604 | scaffold_4 | 27629 | 27745 | 0.75 |
| R33842 | scaffold_15 | 28832 | 28948 | 0.75 |
| R33765 | scaffold_46 | 184730 | 184846 | 0.75 |
| R33543 | scaffold_18 | 419203 | 419319 | 0.75 |
| R33381 | scaffold_12 | 461341 | 461457 | 0.75 |
| R33710 | scaffold_75 | 198231 | 198347 | 0.75 |
| R33853 | scaffold_11 | 442985 | 443101 | 0.75 |
| R33769 | scaffold_44 | 66492 | 66608 | 0.75 |
| R33521 | scaffold_26 | 113519 | 113635 | 0.75 |
| R33472 | scaffold_49 | 33713 | 33829 | 0.75 |
| R35308 | scaffold_14 | 722406 | 722525 | 0.75 |
| R33450 | scaffold_55 | 175307 | 175423 | 0.75 |
| R33491 | scaffold_38 | 290673 | 290789 | 0.75 |
| R33759 | scaffold_48 | 178431 | 178547 | 0.75 |
| R33649 | scaffold_43 | 200653 | 200769 | 0.75 |
| R33536 | scaffold_19 | 117605 | 117721 | 0.75 |
| R33790 | scaffold_31 | 239670 | 239786 | 0.75 |
| R35307 | scaffold_14 | 697379 | 697498 | 0.75 |
| R33405 | scaffold_192 | 28406 | 28522 | 0.75 |
| R33477 | scaffold_47 | 166483 | 166599 | 0.75 |
| R33459 | scaffold_53 | 235254 | 235370 | 0.75 |
| R33409 | scaffold_104 | 1384 | 1500 | 0.75 |
| R33530 | scaffold_20 | 242441 | 242557 | 0.75 |
| R33880 | scaffold_6 | 193666 | 193782 | 0.75 |
| R33498 | scaffold_35 | 126331 | 126447 | 0.75 |
| R33532 | scaffold_20 | 336059 | 336175 | 0.75 |
| R33487 | scaffold_42 | 25622 | 25738 | 0.75 |
| R33776 | scaffold_38 | 270411 | 270527 | 0.75 |
| R33694 | scaffold_98 | 2773 | 2889 | 0.75 |
| R33368 | scaffold_41 | 297689 | 297805 | 0.75 |
| R33681 | scaffold_425 | 614 | 730 | 0.75 |
| R33797 | scaffold_29 | 302267 | 302383 | 0.75 |
| R33552 | scaffold_15 | 339148 | 339264 | 0.75 |
| R33749 | scaffold_51 | 138079 | 138195 | 0.75 |
| R33812 | scaffold_24 | 271390 | 271506 | 0.75 |
| R33424 | scaffold_78 | 56164 | 56280 | 0.75 |
| R33580 | scaffold_7 | 212532 | 212648 | 0.75 |
| R33584 | scaffold_7 | 499753 | 499869 | 0.75 |
| R33676 | scaffold_975 | 9 | 125 | 0.75 |
| R33705 | scaffold_83 | 25572 | 25688 | 0.75 |
| R33436 | scaffold_64 | 179041 | 179157 | 0.75 |
| R33437 | scaffold_64 | 126540 | 126656 | 0.75 |
| R33868 | scaffold_7 | 241889 | 242005 | 0.75 |
| R33505 | scaffold_31 | 373684 | 373800 | 0.75 |
| R33451 | scaffold_55 | 216208 | 216324 | 0.75 |
| R33653 | scaffold_41 | 170328 | 170444 | 0.75 |
| R33673 | scaffold_1454 | 579 | 695 | 0.75 |
| R33398 | scaffold_264 | 8081 | 8197 | 0.75 |
| R33488 | scaffold_42 | 93019 | 93135 | 0.75 |
| R33401 | scaffold_213 | 2016 | 2132 | 0.75 |
| R33512 | scaffold_29 | 275905 | 276021 | 0.75 |
| R33860 | scaffold_9 | 269990 | 270106 | 0.75 |
| R33881 | scaffold_6 | 284333 | 284449 | 0.75 |
| R33522 | scaffold_26 | 189587 | 189703 | 0.75 |
| R33678 | scaffold_690 | 1870 | 1986 | 0.75 |
| R33837 | scaffold_17 | 69351 | 69467 | 0.75 |
| R33531 | scaffold_20 | 117000 | 117116 | 0.75 |
| R33541 | scaffold_18 | 119538 | 119654 | 0.75 |
| R33685 | scaffold_248 | 6695 | 6811 | 0.75 |
| R33432 | scaffold_65 | 50510 | 50626 | 0.75 |
| R33496 | scaffold_36 | 172370 | 172486 | 0.75 |
| R33468 | scaffold_50 | 134152 | 134268 | 0.75 |
| R33763 | scaffold_46 | 171479 | 171595 | 0.75 |
| R33394 | scaffold_528 | 3550 | 3666 | 0.75 |
| R33454 | scaffold_54 | 276314 | 276430 | 0.75 |
| R33794 | scaffold_30 | 52788 | 52904 | 0.75 |
| R33518 | scaffold_27 | 28523 | 28639 | 0.75 |
| R33602 | scaffold_5 | 60822 | 60938 | 0.75 |
| R33561 | scaffold_13 | 103441 | 103557 | 0.75 |
| R33431 | scaffold_65 | 54141 | 54257 | 0.75 |
| R33575 | scaffold_9 | 399400 | 399516 | 0.75 |
| R33582 | scaffold_7 | 413996 | 414112 | 0.75 |
| R33455 | scaffold_54 | 202096 | 202212 | 0.75 |
| R33445 | scaffold_56 | 55855 | 55971 | 0.75 |
| R33406 | scaffold_188 | 1816 | 1932 | 0.75 |
| R33605 | scaffold_4 | 565311 | 565427 | 0.75 |
| R33659 | scaffold_23 | 34885 | 35001 | 0.75 |
| R33428 | scaffold_68 | 145649 | 145765 | 0.75 |
| R33378 | scaffold_23 | 33781 | 33897 | 0.75 |
| R33833 | scaffold_18 | 441037 | 441153 | 0.75 |
| R33542 | scaffold_18 | 88402 | 88518 | 0.75 |
| R33784 | scaffold_33 | 38699 | 38815 | 0.75 |
| R33571 | scaffold_9 | 481854 | 481970 | 0.75 |
| R33669 | scaffold_10 | 272732 | 272848 | 0.75 |
| R33538 | scaffold_18 | 387521 | 387637 | 0.75 |
| R33845 | scaffold_14 | 595005 | 595121 | 0.75 |
| R33870 | scaffold_7 | 462757 | 462873 | 0.75 |
| R33730 | scaffold_56 | 22661 | 22777 | 0.75 |
| R33760 | scaffold_48 | 176246 | 176362 | 0.75 |
| R33562 | scaffold_13 | 280866 | 280982 | 0.75 |
| R33500 | scaffold_33 | 82276 | 82392 | 0.75 |
| R33679 | scaffold_568 | 3511 | 3627 | 0.75 |
| R33376 | scaffold_23 | 208578 | 208694 | 0.75 |
| R33689 | scaffold_202 | 6105 | 6221 | 0.75 |
| R33895 | scaffold_2 | 173590 | 173706 | 0.75 |
| R33778 | scaffold_37 | 186613 | 186729 | 0.75 |
| R33412 | scaffold_93 | 42638 | 42754 | 0.75 |
| R33443 | scaffold_57 | 68332 | 68448 | 0.75 |
| R33366 | scaffold_41 | 300171 | 300287 | 0.75 |
| R33402 | scaffold_202 | 7960 | 8076 | 0.75 |
| R33610 | scaffold_2 | 178984 | 179100 | 0.75 |
| R33549 | scaffold_16 | 173307 | 173423 | 0.75 |
| R33744 | scaffold_52 | 194080 | 194196 | 0.75 |
| R33699 | scaffold_92 | 32704 | 32820 | 0.75 |
| R33813 | scaffold_24 | 71218 | 71334 | 0.75 |
| R33858 | scaffold_9 | 155077 | 155193 | 0.75 |
| R35302 | scaffold_29 | 28962 | 29081 | 0.75 |
| R33802 | scaffold_28 | 29223 | 29339 | 0.75 |
| R33391 | scaffold_745 | 1735 | 1851 | 0.75 |
| R33787 | scaffold_32 | 155532 | 155648 | 0.75 |
| R33494 | scaffold_37 | 317953 | 318069 | 0.75 |
| R33434 | scaffold_65 | 98002 | 98118 | 0.75 |
| R33722 | scaffold_63 | 123612 | 123728 | 0.75 |
| R33479 | scaffold_46 | 124232 | 124348 | 0.75 |
| R21260 | scaffold_11 | 263083 | 263201 | 0.752066116 |
| R9157 | scaffold_9 | 459390 | 459508 | 0.752066116 |
| R33624 | scaffold_136 | 12016 | 12131 | 0.758333333 |
| R33380 | scaffold_12 | 426513 | 426629 | 0.758333333 |
| R33668 | scaffold_10 | 377644 | 377760 | 0.758333333 |
| R33632 | scaffold_67 | 68115 | 68228 | 0.758333333 |
| R33356 | scaffold_203 | 3843 | 3959 | 0.758333333 |
| R33361 | scaffold_43 | 173179 | 173295 | 0.758333333 |
| R33355 | scaffold_912 | 870 | 986 | 0.758333333 |
| R33671 | scaffold_8 | 906898 | 907014 | 0.758333333 |
| R33375 | scaffold_23 | 185205 | 185321 | 0.758333333 |
| R33645 | scaffold_76 | 27622 | 27738 | 0.758333333 |
| R33801 | scaffold_28 | 98083 | 98200 | 0.758333333 |
| R33365 | scaffold_41 | 74139 | 74255 | 0.758333333 |
| R33371 | scaffold_28 | 235476 | 235592 | 0.758333333 |
|  | | | | |
