# Supplementary material for: Comparative Analysis of the 5S rRNA and Its Associated Proteins Reveals Unique Primitive Rather Than Parasitic Features in Giardia lamblia
Source: PLoS One. 2012 Jun 7;7(6):e36878. doi: 10.1371/journal.pone.0036878 (PMC3369914; doi:10.1371/journal.pone.0036878)
Supplement: Table S2 — The identified C2H2-type zinc finger proteins in G. lamblia WB genome. (DOC) [file pone.0036878.s002.doc]

**Table S2. The identified C2H2-type zinc finger proteins in *G. lamblia* WB genome.**

| **ORF** | **Number of C2H2 repeats** | **Potential functions** |
| --- | --- | --- |
| 10033 | 1 | Hypothetical protein |
| 10755 | 2 | Splicing factor 3A subunit 2 |
| 113416 | 1 | High cysteine membrane protein |
| 114180 | 1 | High cysteine membrane protein TMK-like |
| 11692 | 1 | VSP, putative |
| 13464 | 1 | Splicesome-associated protein |
| 137673 | 2 | Reverse transcriptase/endonuclease, putative |
| 14069 | 2 | Hypothetical protein |
| 16300 | 1 | Protein kinase |
| 16406 | 2 | Chaperone protein |
| 17003 | 4 | Zinc finger protein |
| 17480 | 1 | tRNA delta2-isopentenylpyrophosphate transferase |
| 19815 | 1 | Hypothetical protein |
| 27035 | 1 | Hypothetical protein |
| 3763 | 1 | Hypothetical protein |
| 5822 | 1 | probable membrane protein |
| 8405 | 1 | Hypothetical protein |
| 8920 | 1 | Zinc finger protein |
|  | | |
