# Supplementary material for: Comparative Analysis of the 5S rRNA and Its Associated Proteins Reveals Unique Primitive Rather Than Parasitic Features in Giardia lamblia
Source: PLoS One. 2012 Jun 7;7(6):e36878. doi: 10.1371/journal.pone.0036878 (PMC3369914; doi:10.1371/journal.pone.0036878)
Supplement: Table S3 — Accession numbers of genes of transcriptional factors and specific subunits of RNA pol III in G. lamblia, T. vaginalis, T. cruzi, and N. gruberi. (DOC) [file pone.0036878.s003.doc]

**Table S3. Accession numbers of genes of transcriptional factors and specific subunits of RNA pol III in *G. lamblia*, *T. vaginalis, T. cruzi,* and *N. gruberi***.

|  | | ***G. lamblia*** | | | ***T. vaginalis*** | ***T. cruzi*** | ***N. gruberi*** |
| --- | --- | --- | --- | --- | --- | --- | --- |
|  | | **WB** | **GS** | **P15** |  |  |  |
| **TFIIIA** | |  |  |  | TVAG_458980 |  |  |
| **TFIIIB** | **TBP** | GL50803_1721 | GL50581_2180 | GLP15_4095 | TVAG_291560 | Tc00.1047053503809.14909.149 | Naegr1_78851 |
|  | **BRF** | GL50803_4125 | GL50581_3049 | GLP15_4829 | TVAG_139840 | Tc00.1047053507093.180 | Naegr1_61196 |
|  | **B’’** |  |  |  | TVAG_482180 | Tc00.1047053503779.50 | Naegr1_78613 |
| **TFIIIC** | **TFIIIC 102** |  |  |  | TVAG_324710 | Tc00.1047053506529.490 | Naegr1_79599 |
|  | **TFIIIC 63** |  |  |  | TVAG_186100 | Tc00.1047053506855.370 | Naegr1_57146 |
| **RNA pol III specific subunits** | **C34** | GL50803_121062 | GL50581_1196 | GLP15_2637 | TVAG_074910 | Tc00.1047053503685.2085.20 | Naegr1_29241 |
|  | **C53** |  |  |  | TVAG_458550 | Tc00.1047053508461.110 | Naegr1_77856 |
|  | **C82** | GL50803_4155 | GL50581_1430 | GLP15_3091 | TVAG_156540 |  | Naegr1_66106 |
|  | **C17** |  |  |  |  |  | Naegr1_78611 |
