# Supplementary material for: Comparative Analysis of the 5S rRNA and Its Associated Proteins Reveals Unique Primitive Rather Than Parasitic Features in Giardia lamblia
Source: PLoS One. 2012 Jun 7;7(6):e36878. doi: 10.1371/journal.pone.0036878 (PMC3369914; doi:10.1371/journal.pone.0036878)
Supplement: Table S4 — Comparisons of the 5S rRNA system between G. lamblia and Encephalitozoon cuniculi. (DOC) [file pone.0036878.s004.doc]

# Table S4. Comparisons of the 5S rRNA system between *G. lamblia* and *Encephalitozoon cuniculi*.

|  | | | ***G. lamblia*** | | | ***E. cuniculi*** |
| --- | --- | --- | --- | --- | --- | --- |
|  | | | **WB** | **GS** | **P15** |  |
| **5S rRNA gene** | **Copy number** | | **8 ^[16]^** | **Y^*^ (?)** | **6 ^[21]^** | **3^*^** |
|  | **Tandem arrangement** | | **N** | **N^*^** | **N** | **N^*^** |
|  | **Located with rDNA Units** | | **N** | **N^*^** | **N** | **N^*^** |
| **Promoter** | **ICR** | | **N^*^** | **N^*^** | **N^*^** | **Y^*^** |
|  | **TATA-box** | | **Y^*^** | **N^*^** | **N^*^** | **Y^*^** |
| **Transcription factor** | **TFIIIA** | | **N** | **N^*^** | **N^*^** | **N^*^** |
|  | **TFIIIB** | **TBP** | **Y ^[17]^** | **Y^*^** | **Y^*^** | **Y^*^ (ECU04_1440)** |
|  |  | **TFB/BRF** | **Y ^[17]^** | **Y^*^** | **Y^*^** | **Y^*^ (ECU05_0500)** |
|  |  | **B’’** | **N** | **N^*^** | **N^*^** | **Y^*^ (ECU03_1040)** |
|  | **TFIIIC** | **TFIIIC 102** | **N** | **N^*^** | **N^*^** | **Y^*^ (ECU08_0840)** |
|  |  | **TFIIIC 63** | **N** | **N^*^** | **N^*^** | **Y^*^ (ECU08_1770)** |
| **RNA pol III specific subunit** | **C34** | | **Y ^[17]^** | **Y^*^** | **Y^*^** | **Y^*^ (ECU10_0210)** |
|  | **C82** | | **Y^*^** | **Y^*^** | **Y^*^** | **Y^*^ (ECU09_0250)** |
|  | **C31** | | **N** | **N^*^** | **N^*^** | **N^*^** |
|  | **C17** | | **N** | **N^*^** | **N^*^** | **N^*^** |
|  | **C53** | | **N** | **N^*^** | **N^*^** | **N^*^** |
| **L5 protein** | | | **Y** | **Y** | **Y** | **Y** |
